# *In situ* simulation for trauma victim care in a hospital fire scenario

**DOI:** 10.31744/einstein_journal/2026AO2275

**Published:** 2026-06-23

**Authors:** Marcia Bucco, Juliana Ollé Mendes, Jorge Vinícius Cestari Felix

**Affiliations:** 1 Universidade Federal do Paraná Curitiba PR Brazil Universidade Federal do Paraná, Curitiba, PR, Brazil.

**Keywords:** Simulation training, Fires, Disasters, Education, continuing, Hospitals, Wounds and injuries, Safety, Patient safety

## Abstract

**Objective:**

To evaluate *in situ* simulations as a training strategy for the initial care of trauma victims in a hospital fire scenario.

**Methods:**

A qualitative, descriptive, and exploratory study was conducted using *in situ* simulations, following the Consolidated Criteria for Reporting Qualitative Research (COREQ). It was developed in a large hospital in the capital of Paraná with the participation of 16 professionals. Data analysis was conducted using Bardin's Content Analysis and categories were evaluated based on Kolb's theoretical framework for experiential learning.

**Results:**

The responses were categorized into six categories, which allowed us to highlight simulation as a qualification strategy for professionals working in hospitals during disasters.

**Conclusion:**

In situ simulations demonstrated the potential for training in critical situations, promoting multi-professional integration, strengthening safety culture, and identifying weaknesses in institutional protocols. Periodic inclusion of this type of training in hospital safety plans is recommended.

## In Brief

This study was conducted in a large tertiary hospital to evaluate in situ simulation as a training strategy for the initial care of trauma victims during a hospital fire scenario. The simulation involved 16 healthcare professionals and demonstrated that through in situ training, teams become more qualified for critical situations. Analysis of participants' experiences revealed six key categories: improved professional preparedness, multi-professional integration, strengthened safety culture, and the identification of gaps in institutional protocols. These findings support the periodic inclusion of in situ simulations in hospital safety and emergency preparedness plans.

## Highlights

■ *In situ* simulation prepares healthcare teams for hospital fire emergencies.
■ Simulation strengthens teamwork, communication, and safety culture.
■ This study demonstrates that simulation enables the identification of latent threats and gaps in institutional protocols.
■ *In situ* training contributes to organizational readiness and emergency planning.

## Graphical abstract

**Figure:**
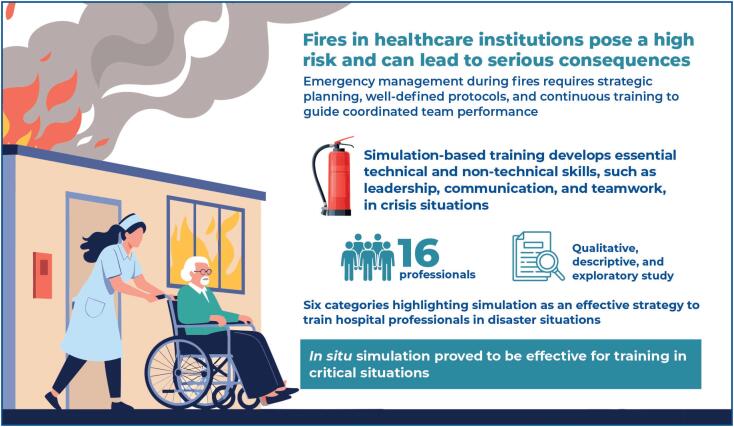

## INTRODUCTION

Hospital safety is an essential aspect of quality of care and patient safety, especially in critical situations such as hospital fires, which pose serious threats to the lives of patients, professionals, and visitors. Although such events are rare, fires in health institutions pose a high risk and may result in catastrophic consequences when teams are not adequately trained or there are no standardized protocols for such responses.^(1)^

In this sense, the Permanent Health Education (PHE) has assumed a strategic role through the continuous development of practices, including updating professionals in response to technological, scientific and organizational changes in health systems.^(2,3)^

This approach, established by the National Continuing Health Education Policy (PNES) in 2004,^(3)^ integrates teaching and service into an educational process that starts from the real needs of practice and is aimed at improving teamwork and quality of care.

Thus, emergency management in fire situations requires strategic planning, well-defined protocols and continuous training that aim to guide the synchronized performance of the team. In this sense, simulation training is a strategy for the development of technical and non-technical skills, such as leadership, communication, and teamwork, which are essential during crisis situations.^(4)^

To this end, clinical simulation (CS) has been widely used in the formation of multiprofessional teams because it enables the improvement of self-confidence and decision-making under pressure, promotes interprofessional integration, and strengthens safety culture.^(5,6)^ Therefore, of the different existing modalities, *in situ* simulation (ISS) stands out because it is being performed in the actual work environment, providing realism and contextualization to the training. Furthermore, it allows professionals to experience complex situations under controlled conditions, and to recognize system weaknesses, knowledge gaps, and structural challenges that could compromise the response in a real event.^(7- 9)^

Thus, this study was based on the theory of experiential learning developed by Kolb^(10)^ in which knowledge is created through the transformation of experience, constituting a process of transformation that is continuously created and recreated.

## OBJECTIVE

To evaluate *in situ* simulation as a training strategy for the initial care of trauma victims in a hospital fire scenario.

## METHODS

### Study design

Qualitative, descriptive, and exploratory studies using ISS were used as strategies for PHE. The Consolidated Criteria for Reporting Qualitative Research (COREQ)^(11)^ were adopted to increase research rigor and quality. Thus, we sought to understand the perceptions and experiences of professionals who participated in the simulation.

### Population

ISS was established in October 2024 at a large philanthropic hospital located in the capital of Paraná. This institution serves cases of low, medium, and high complexity, with an average of 17,000 admissions annually. Six brigade members and three members of the Rapid Response Team (RRT), comprising medical and nursing staff, participated in the simulation. In the Emergency Room (ER), there were seven professionals: one nurse, two doctors, and four nursing technicians.

Participants were selected by convenience sampling. The inclusion criteria were as follows: professionals aged between 18 and 60 years, active in the institution during the research period, who agreed to participate voluntarily in the simulations and debriefing after reading and signing an Informed Consent Form (ICF). Professionals who were on vacation, leave, or absent during the study period were excluded from the study.

To this end, the professionals were identified by sequential coding, using the letter "P" for the professionals, followed by Arabic numbers, ensuring anonymity.

### Data collection

An ISS was planned for an accident involving electrical wiring that resulted in a hospital fire. The scenario began with a maintenance worker who suffered an electric shock from a short circuit during an intervention in the electrical network, followed by a fall of approximately 1.5 m. The event caused facial trauma and an exposed fracture in the lower right limb; therefore, a moulage was used.

**Figures 1:**
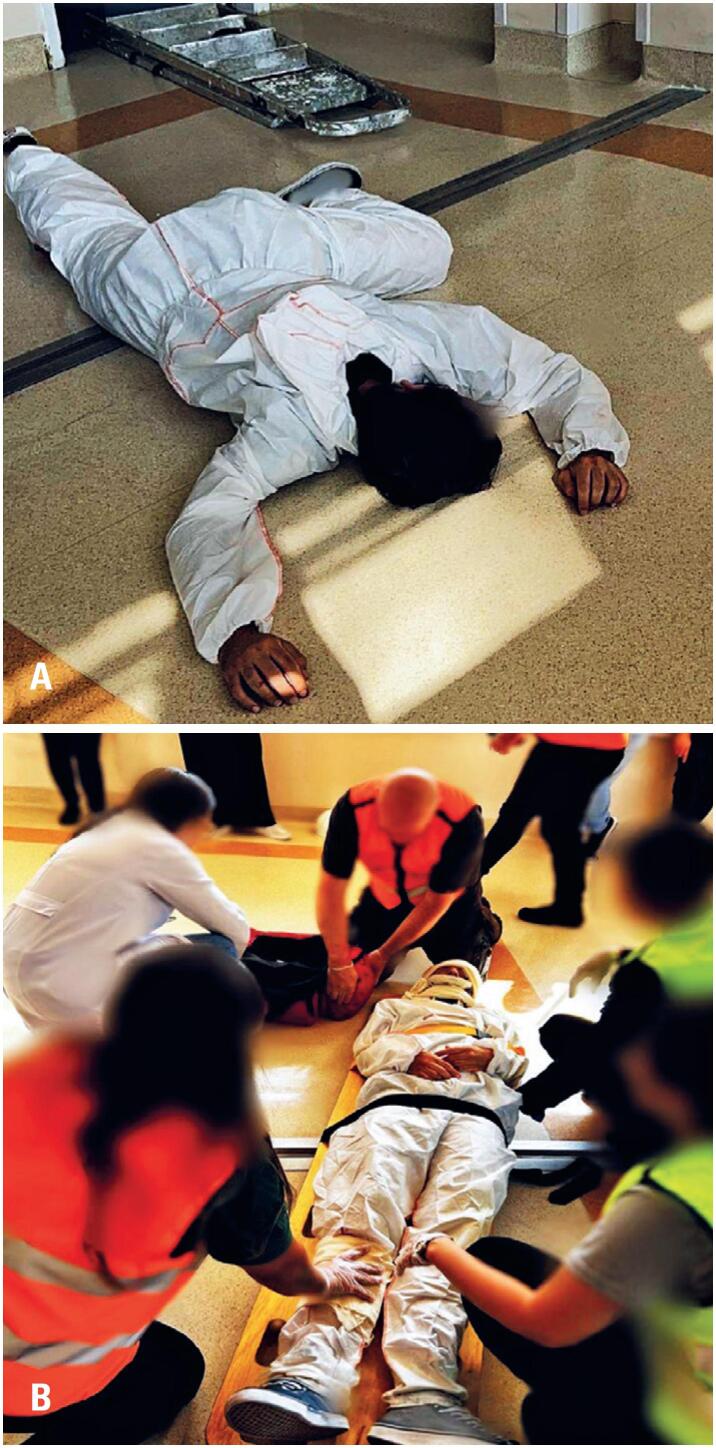
(A–B) simulated scenario and the professionals’ actions during the service

There was a need for an immediate response from the teams, requiring the containment of the fire and care for trauma victims. The simulated scenario was conducted on hospital premises and lasted 10 min, the time required for the containment of the fire focus, the care of the simulated victim, and the removal of the victim from the site. The main stages of the simulation and dynamics are shown in figures 1A –B.

This simulation was supported by the Civil Defense of the municipality and the direction of the institution through a prior agreement. The event was publicized 15 days in advance through institutional posters, internal communications, and social networks. The ISS was observed by trained facilitators, followed by a structured debriefing lasting approximately 20 min.

Data collection occurred soon after the completion of the simulated scenario in a room isolated from the care area and a semi-structured questionnaire was used to evaluate ISS as a training strategy for the initial care of trauma victims in a hospital fire scenario.

The responses were recorded and later transcribed by the researchers, being stored through a document of the software Microsoft Office Word^®^, coded. Subsequently, a descriptive analysis of the participants’ characteristics and content analysis were conducted.^(12)^

### Data analysis

Content analysis by Bardin,^(12)^ used in qualitative studies, seeks to interpret meanings, perceptions, and experiences expressed in discourses. The analytical process took place in three interdependent stages: pre-analysis, with a floating reading of the transcribed material and organization of the textual corpus; exploration of the material by identifying the units of registration and grouping the lines into thematic categories; and treatment of the results and interpretation, which establishes the synthesis of the main categories and the construction of inferences.

### Ethical aspects

This study followed the guidelines established by Law No. 14.874 of May 28, 2024, of the National Health Council (CNS), through the presentation of the research and provision of an ICF to all participants. This study was approved by the Research Ethics Committee of the *Universidade Federal do Paraná,* CAAE: 77371323.8.0000.0102; # 6.826.084, and *Secretaria Municipal da Saúde de Curitiba,* CAAE: 77371323.8.3001.0101; # 6.958.554.

## RESULTS

The sample consisted of 16 professionals, including 10 female participants (62.5%) and 6 male participants (37.5%). Concerning academic training, one participant (6.25%) had a master's degree, and three (18.75%) were experts in their working areas. The remaining 12 professionals (75%) had technical training or had fully graduated.

In relation to the distribution by profession, six participants (37.5%) were brigade members responsible for the initial actions to contain the fire and removal of patients from the site; three participants (18.75%) were part of the Rapid Response Team composed of doctors and nurses; and seven participants (43.75%) belonged to the Emergency Room (ER), including one nurse (6.25%), two doctors (12.5%), and four nursing technicians (25%).

After the sociodemographic and functional characterization of the participants, a qualitative analysis of their answers to the open questions was conducted after debriefing. The textual material consisting of the narratives of the professionals involved was submitted for content analysis according to Bardin.^(12)^

The main categories reflected dimensions related to the learning of professionals, development of technical and non-technical skills, and the perception of simulation as a strategy for strengthening patient safety culture. The following synthesis categorizes responses regarding the perception of the effectiveness of ISS for trauma victim care training in hospital fire scenarios (Table 1).

**Table 1:** Main thematic categories regarding the perception of the effectiveness of *in situ* simulation

Category	Description	Examples of citations
Professional training	Recognizing simulation as a strategy in technical and emotional preparation.	"Simulation is of utmost importance for preparing and training healthcare professionals." "It's important so that the entire team knows how to act."
Developing non-technical skills	Emphasis on communication, teamwork, leadership, and emotional control.	"The simulation provided insight into how we should act under pressure." "Extremely important, as it highlighted the need for effective communication."
Identifying shortcomings and opportunities for improvement	Using a simulation to identify institutional vulnerabilities.	"It was crucial for identifying flaws in the customer service process." "It's important for finding areas for improvement."
Practical learning	Practical learning, skills reinforcement, and conduct review.	"The simulation allowed us to learn from mistakes in a controlled environment." "It was essential for identifying flaws in the service process."
Patient safety and organizational preparedness	Direct link between simulation and healthcare safety.	"Simulations like this make the hospital environment safer." "It strengthened my sense of urgency and collaboration."
Permanent education	Suggestion to formalize the periodic simulations.	"Training like this should be ongoing, not just a one-off event." "I believe all professionals should undergo this type of training."

## DISCUSSION

The results show that professionals considered ISS an important strategy for technical, cognitive, and emotional training in trauma and hospital fire scenarios. The categories highlighted its role in training, integrating theory and practice, identifying failures, developing non-technical skills, promoting patient safety, and strengthening continuing education.

The category "professional training" directly aligns with the scoping review of Bucco et al.,^(1)^ which mapped the international evidence on the use of simulations in hospital fires and concluded that simulation training is crucial to prepare teams for critical events, promoting coordination, communication and care safety. The authors emphasize that, although rare, hospital fires have a high potential for severity, and simulation is one of the most effective ways to reproduce realistic scenarios and evaluate the readiness of teams in case of a fire emergency.

In the present study, the participants’ responses reinforce this perception by describing the simulation as "essential for the whole team to know how to act." These data align with the results of Bucco et al.^(1)^ who found that learning using simulation favors fast and safe responses. These data corroborate the reports of the participants when they mentioned that every team needs to know how to act.

The category "developing non-technical skills" is also supported by evidence from Sarwani et al.,^(13)^ whose scoping review on interprofessional simulations in the surgical center showed that *in situ* training improves communication, leadership and cooperation between multidisciplinary teams, reducing morbidity and mortality and increasing patient safety.

The category "identifying shortcomings and opportunities for improvement" reveals simulation as a strategy that enables the diagnosis of security threats. This finding is corroborated by Katz-Dana et al.^(14)^ who used ISS to test clinical systems in a newly opened emergency room and identified 113 latent threats to health (LTH). This strategy successfully identified security threats and improved team preparedness, which may be used to guide future operational plans. The present study demonstrated similar results, in which professionals recognized that simulation was "fundamental to identifying failures in the service process," confirming the potential of the ISS to improve processes and prevent risks before they become real events.

Another notable aspect concerns the motivational and reflexive dimension of learning, associated with the category "practical learning." The participants’ perceptions regarding simulation as a means of "learning from mistakes in a controlled environment" are supported by Henrique-Sanches et al.,^(15)^ whose review on clinical simulation and motivation in nursing and medical learning demonstrated that simulated living increases motivation, self-confidence and critical thinking, as well as reducing anxiety. These effects are explained by Kolb's experiential learning theory^(10)^ in which knowledge is created through the transformation of experience, which is reflected in the pedagogical structure of the analyzed hospital simulations.

The relationship between simulation and "patient safety and organizational preparedness," expressed in the participants’ responses such as "simulations like this make the hospital environment safer," is in agreement with the findings of Diebel et al.,^(16)^ who applied ISS in rural emergency services in Canada and observed an increase in confidence in the management of acute situations, as well as in the satisfaction and safety perception of professionals contributing to improvements in patient care. These authors highlight that ISS is viable even in contexts with few resources because it is well structured, which reinforces its adaptability and potential for dissemination at different levels of hospital complexity.

Finally, the category "Permanent Education," in which professionals suggested to formalize periodic training, confirms the strategic role of ISS and the PHE strategy. This perspective was corroborated by Bucco et al.^(1)^ and Sarwani et al.^(13)^ who advocated the systematic incorporation of ISS in quality improvement and hospital safety cycles. Therefore, the evidence identified in this study indicates that the continuity of training is essential to improve skill development and consolidate institutional safety culture.

As a limitation, it should be noted that because this was a study with a qualitative approach and descriptive character, the small number of participants and the realization of a single ISS restricted more comprehensive inferences about the subject.

## CONCLUSION

The results of this research reinforce the role of *in situ* simulations as a strategy in Permanent Health Education, enabling the identification of institutional vulnerabilities aimed at the optimization of care flows and alignment of professional practice to hospital safety policies. *In situ* simulations presents itself as an experiential and reflexive learning strategy, supporting Kolb's theory of transforming experience into knowledge. Therefore, it is recommended to conduct new studies using *in situ* simulations in other disaster scenarios and periodically include this type of training in hospital safety plans.

## Data Availability

The underlying content is contained within the manuscript.
